# A single acute alcohol intoxication before fracture insult causes long-term elevated systemic RANKL and OPG levels in young adult mice

**DOI:** 10.1038/s41598-025-09240-3

**Published:** 2025-07-08

**Authors:** Katrin Bundkirchen, Weikang Ye, Tianqi Zhang, Friederike Weidemann, Stefan Lienenklaus, Bastian Welke, Helen Rinderknecht, Borna Relja, Claudia Neunaber

**Affiliations:** 1 Hannover Medical School, Department of Trauma Surgery, Hannover, Germany; 2 Ulm University Medical Center, Department of Trauma, Hand, Plastic and Reconstructive Surgery, Translational and Experimental Trauma Research, Ulm, Germany; 3 Hannover Medical School, Institute of Laboratory Animal Science, Hannover, Germany; 4 Hannover Medical School, Department of Orthopaedic Surgery, Laboratory for Biomechanics and Biomaterials, Hannover, Germany

**Keywords:** Ethanol, Bone regeneration, Hemorrhagic shock, µCT, Histology, Biomechanics, Osteoblasts, Osteoclasts, Osteocytes, Bone, Cartilage, Trauma, Nutrition disorders, Bone imaging, Radiography, Three-dimensional imaging, Nutrition, Inflammation, Risk factors

## Abstract

Binge drinking is the most common form of alcohol abuse and 25–40% of orthopedic trauma patients are intoxicated upon admission. While alcohol consumption prolongs the healing time of bone fractures, the underlying mechanisms by which alcohol leads to this fracture healing disorder remain unclear. 240 young adult and aged male C57BL/6J mice were examined. Half of the animals were acutely alcoholized two hours before surgery. The isolated fracture (Fx) group underwent an externally stabilized osteotomy and another group (THFx) an additional trauma hemorrhage (TH). The Sham group only received catheterization and fixation. Histological, radiological, biomechanical and systemic examinations were performed. Data were analyzed using two-way ANOVA and *p* ≤ 0.05 was considered significant. After 3 weeks, acute alcoholization led to increased receptor activator of NF-κB ligand (RANKL), osteoprotegerin (OPG) and RANKL/OPG ratio after fracture in young adult mice. In aged animals, this effect of alcohol was not present as values were already increased in the sober ones. More osteoblasts and macrophages were found in alcoholized young Fx animals. In conclusion, one-time binge drinking before trauma causes a long-term change in the RANKL/OPG ratio towards more RANKL in young adult mice.

**Supplementary Information:**

The online version contains supplementary material available at 10.1038/s41598-025-09240-3.

## Introduction

Alcohol consumption is a significant risk factor for bone fractures. Consumption of ≥ 2 alcoholic drinks per day has been shown to increase the overall risk of fracture and people who regularly consume alcohol have a four times higher risk of fracture than non-drinkers of the same age^1,2^. Overall, 25–40% of orthopaedic trauma patients are acutely intoxicated with alcohol upon admission and retrospective data revealed that 6.1% of all acute fracture admissions were alcohol-related with more than 70% of these patients being male, showing a bimodal distribution peaking between the ages of 20–50 and 60–70^2,3^. The majority of alcohol-related fractures is a result of simple mechanical falls, frequently caused by impaired coordination and balance due to intoxication, and usually leads to fractures of the lower limb^4^. Alarmingly, 51% of young adults are intoxicated with alcohol upon admission to emergency departments after trauma, with binge drinking being the predominant pattern at 78%^3^. Binge drinking is defined as consuming four of more drinks within 2 h for women and five or more drinks for men, resulting in a blood alcohol concentration of around 80 mg/dl^5,6^. Globally, this form of alcohol consumption is the most prevalent, especially among the younger demographics, and is associated with an increase in morbidity and mortality, constituting a substantial public health concern^6^. Furthermore, alcohol-related fractures impose a significant financial burden on the healthcare system due to increased medical interventions and prolonged recovery times^4^.

Despite the established knowledge that alcohol consumption constitutes a risk factor for orthopedic trauma and contributes to comprised bone health, the relationship between alcohol consumption and fracture healing remains to be elucidated^1,7^. Especially in younger patients, the presence of alcohol excess from low-risk to chronic drinking is considered a significant predictive factor for a non-union^1^. A factor that may contribute to the progress of non-union formation is fracture healing time, which has been shown to be significantly prolonged in patients who abuse alcohol^8^.

A number of experimental animal studies on the impact of recurrent binge drinking periods on fracture healing have demonstrated that this pattern of alcohol consumption results in adverse outcomes including diminished fracture callus volume, reduced bone and cartilage formation, as well as poorer biomechanical properties^9–14^. At the cellular level, studies further suggest that the delayed fracture healing process in chronic consumers is among others attributed to an inhibition of osteoblast proliferation^1^.

Receptor activator of NF-κB ligand (RANKL) and osteoprotegerin (OPG) have been shown to play crucial roles in the bone homeostasis and the fracture healing process due to their involvement in bone remodeling and osteoclast regulation. According to our literature review, nothing is known about the effect of binge alcohol drinking combined with fracture on the RANKL – OPG system. Chronic alcohol consumption has been shown to increase osteoclasts ratios meanwhile reducing osteoblasts in an age-dependent manner in rats^15^. The same study also showed an alcohol-induced increase in RANKL expression in trabecular bone in young and aged rats with a more pronounced effect in the older animals, and a concomitant substantial elevation in plasma OPG in young animals following alcohol treatment, a change that remained undetectable in aged rats. However, OPG levels increased significantly in sober old rats.

Unlike most other studies that focus on repeated binge drinking or chronic alcohol abuse, this study investigated how a single acute alcohol intoxication, a common model for immunological and inflammatory issues^16–23^, affects fracture healing. The binge alcohol consumption was administered two hours prior to the induction of trauma consisting of a diaphyseal femur fracture with or without severe blood loss. Local fracture healing was assessed histologically, radiologically and biomechanically in young adult and aged mice in both the short- and long-term. In addition, systemic markers of bone remodeling were assessed.

The following hypotheses were postulated in context of the study:

1) The single binge alcohol dose leads to increased numbers of neutrophils, macrophages and osteoclasts in fractures of young adult mice in the early healing phase (up to 24 h). However, due to the age and the resultant elevated baseline inflammatory status, this effect does not manifest in older animals.

2) (a) Three weeks after fracture, previously alcoholized young adult mice show higher systemic β-Crosslaps (β-CTX), RANKL and OPG levels, with no change in the RANKL/OPG ratio. Again, due to the age and the resultant elevated baseline inflammatory status, this effect does not manifest in older animals. (b) One time alcohol binge does not significantly influence the fracture healing process of both ages analyzed by biomechanical, µCT nor histological parameters.

3) The increased severity of the initial trauma, caused by an additional severe blood loss, exacerbates the negative effects of alcohol on fracture healing.

## Results

### Survival rate and physiologic response

For this study, a total of 274 animals were used. 34 animals died or had to be excluded from the experiment (12.41%), resulting in a total of 240 animals for analysis with *n* = 8 animals per group in the 24 h and *n* = 12 per group in the three week analysis time points.

18 mice died during the experiment (6.57%). Seven animals had to be sacrificed prematurely due to complications or reaching the termination criteria (2.55%). Apart from that, nine mice had to be excluded due to anomalous bone sample results (3.28%). For further information see Supplementary Table 1 (online).

One bone for the histological evaluation at three weeks disintegrated during storage in 70% ethanol and was therefore excluded, resulting in a sample size of *n* = 5 in the Fx-group with ethanol for this analysis.

Due to the unstable bridging of the fracture in bones harvested 24 h after trauma, samples often disintegrated after explantation of the external fixator. As a result, fewer than eight sections per analysis were often evaluated (see graphics).

Alcohol concentrations were measured in the blood of both six young and old animals two hours post intoxication, with an overall value of 143.93 mg/dl. Interestingly, young animals showed with 160.52 mg/dl higher levels then the older animals with only 127.34 mg/dl.

**Fig. 1:**
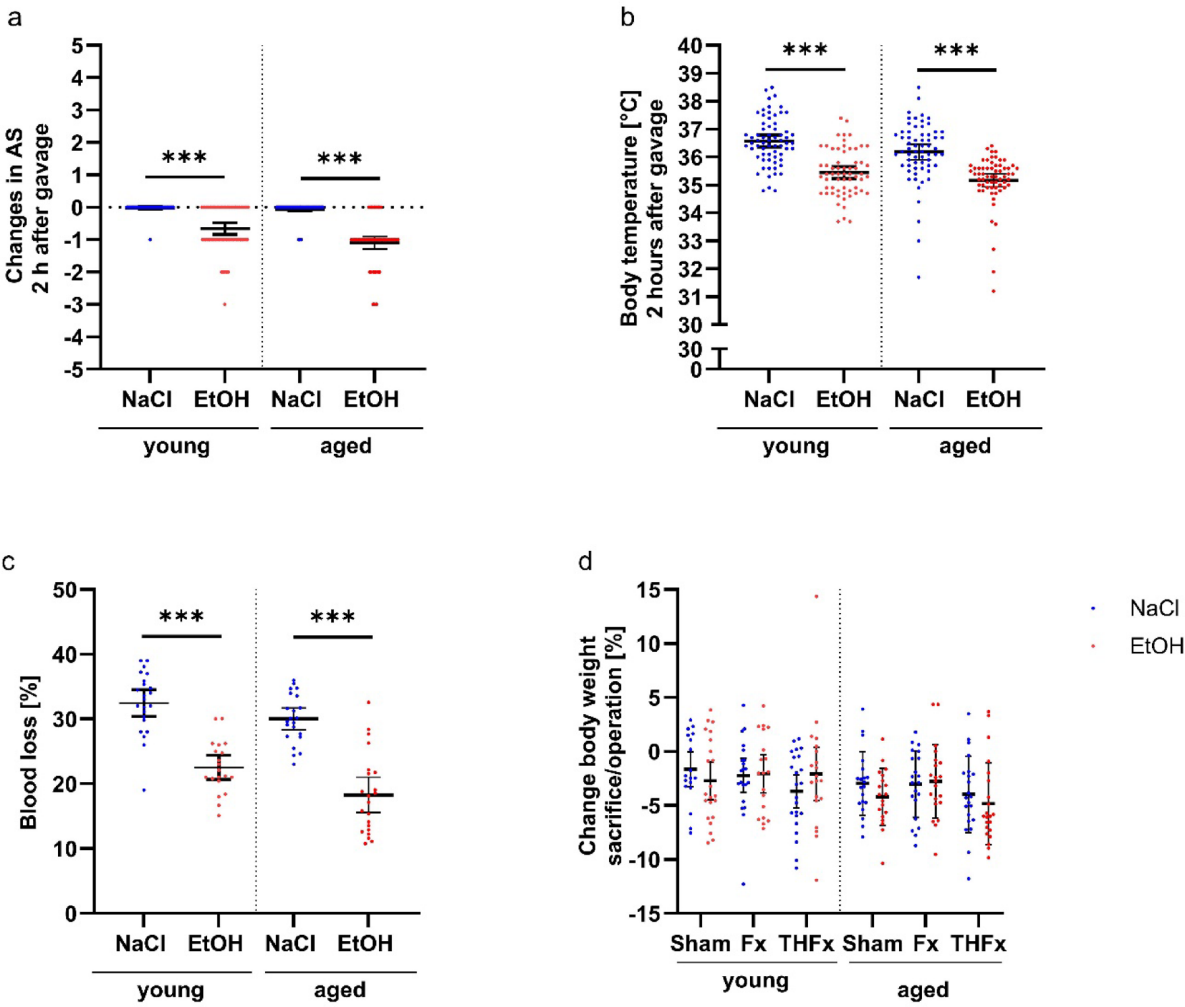
Analysis of physiological parameters. Two hours after alcohol intake, both young and aged animals showed a reduced Activity Score (AS) (**a**) and lower temperature (**b**) compared to mice without alcohol intoxication. Less blood could be collected from drunken young and aged mice in contrast to the sober counterparts (**c**). The change in the body weight did not differ significantly between the operation groups (**d**). Score data as not normally-distributed values were analyzed via Mann-Whitney U Test. The parameters blood loss and change body weight showed normal distributed parametric data and were analyzed with an unpaired t test for the blood loss and by two-way ANOVA for the body weight. ***p < 0.001 vs. indicated group.

The effect of alcohol on changes in the activity score (AS) and the body temperature two hours after gavaging, as well as the percentage of blood withdrawn during shock in the THFx groups were examined independently from the different traumata (Fig. 1a, b, c). Acute alcohol intoxication resulted in a significant decline in the AS compared to animals gavaged with only NaCl in both age groups (*p* < 0.001 for both). Body temperature was also significantly lower in the drunk animals then in the sober ones two hours after administration, regardless of age (*p* < 0.001 for both). In line, less blood could be drawn from previously ethanol intoxicated animals of both age groups (*p* < 0.001 for both).

No significant effects of the alcohol treatment or trauma on the body weight before operation/sacrifice [%] in either young or elderly mice were observed (Fig. 1d).

### Histological analyses

#### Bone cells

**Fig. 2:**
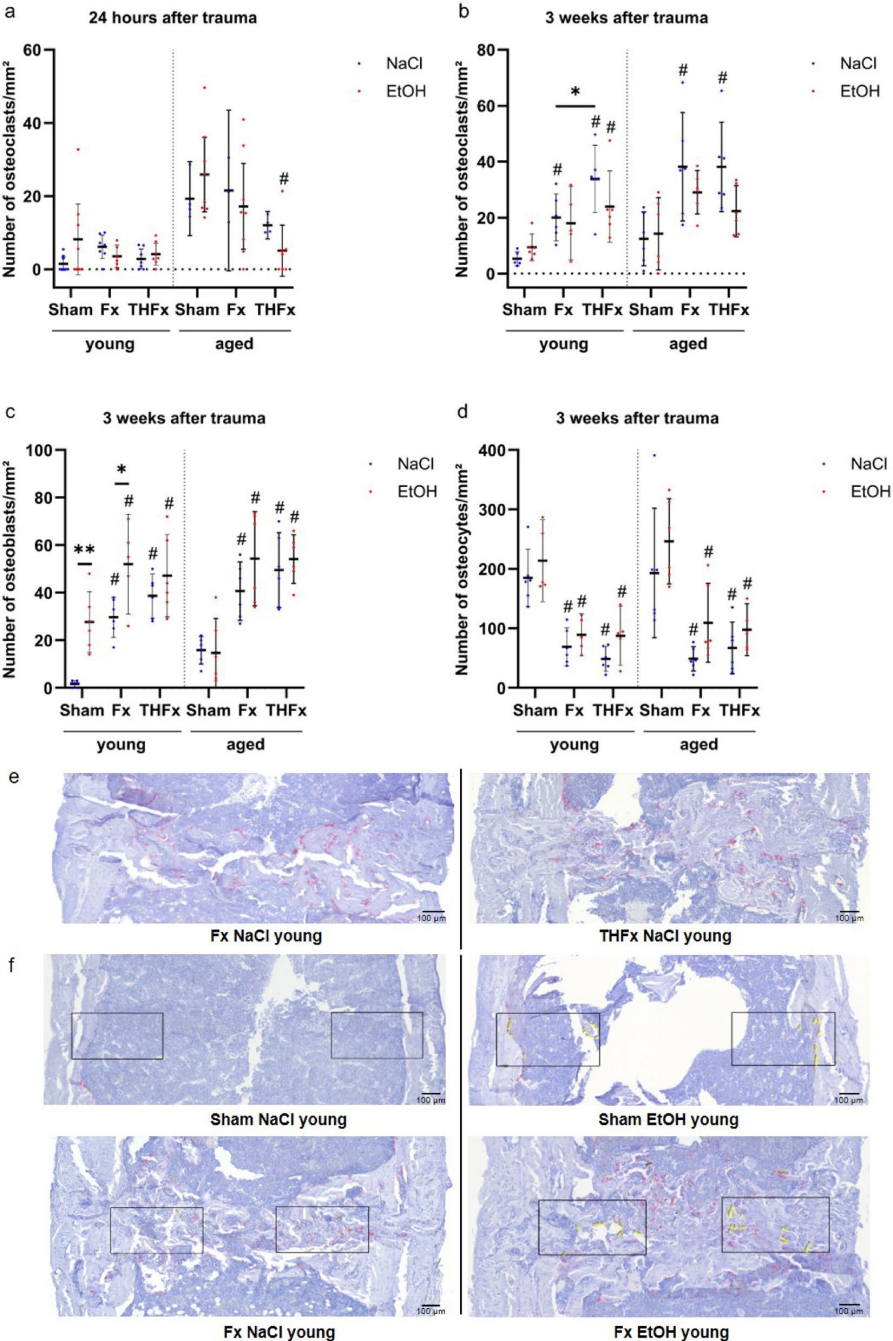
Local analyses of bone cells. (**a**) Alcohol did not cause differences in the number of osteoclasts after 24 h. (**b**) An additional hemorrhagic shock led to more osteoclasts after three weeks in young animals in comparison to the Fx group or three weeks. (**c**) In contrast to sober animals, drunken young Sham and Fx mice showed higher numbers of osteoblasts in the fracture gap three weeks after trauma. For osteocytes (**d**) no effects of alcohol consumption occurred. (**e**) More red stained cells, with bone contact, interpreted as osteoclasts in the TRAP staining, appeared in the young sober THFx groups than in the respective Fx animals after three weeks. (**f**) Significant changes in the numbers of osteoblasts after three weeks: violet, cubic cells arranged in lines with contact to bone, counted as osteoblasts in ImageJ, are marked with crosshairs and yellow numbers. Scale bar 100 μm, magnification 300-fold. * *p* < 0.05 vs. indicated group, # *p* < 0.05 vs. respective Sham. (A) *n* = 3–8, (B) *n* = 5–6, (C) *n* = 5–6, (D) *n* = 5–6 per group.

Alcohol consumption did not lead to significant differences in osteoclast numbers in the fracture gap or the representative area in the Sham groups 24 h after trauma in young and aged mice. Although, a significant change occurred between the different traumata in the elderly mice (*p* = 0.014) and the combined procedure of severe hemorrhage and fracture in old mice reduced the number of osteoclasts 24 h after surgery compared to Sham animals of the same age as shown in Fig. 2a. Similarly, after three weeks, different trauma procedures affected the number of osteoclasts (*p* < 0.001 young and aged) whereas alcohol intoxication did not. In more detail, a significant increase in the osteoclast numbers caused by additional severe blood loss was detectable in young sober mice in comparison to young animals without heavy bleeding (adj. *p* = 0.031, Fig. 2e). This effect was not found in alcoholized mice or the elder population. Moreover, significantly increased osteoclast numbers were detected in contrast to the respective Sham groups as indicated (Fig. 2b).

Osteoblast numbers three weeks after the intervention (Fig. 2c) changed significantly according to type of gavage (NaCl or Ethanol) in young animals (*p* < 0.001) and according to the trauma type in both age groups (*p* < 0.001 for both). Acute ethanol intoxication resulted in significantly more osteoblasts in the fracture gap of young mice three weeks after isolated fracture compared to sober animals (adj. *p* = 0.039, Fig. 2f). The same effect was observed in the young Sham group (adj. *p* = 0.007, Fig. 2f), however not in the group with additional severe blood loss. Described effects were absent in aged mice. Significantly higher amounts of osteoblasts were found in all fractured bones of both young and old mice when compared to the respective Sham groups.

Osteocyte numbers after trauma did not change in regard to alcohol intoxication in both age groups, but due to different traumata (*p* < 0.001 for both) compared to the respective Sham groups as displayed in Fig. 2d.

#### Innate immune cells

**Fig. 3:**
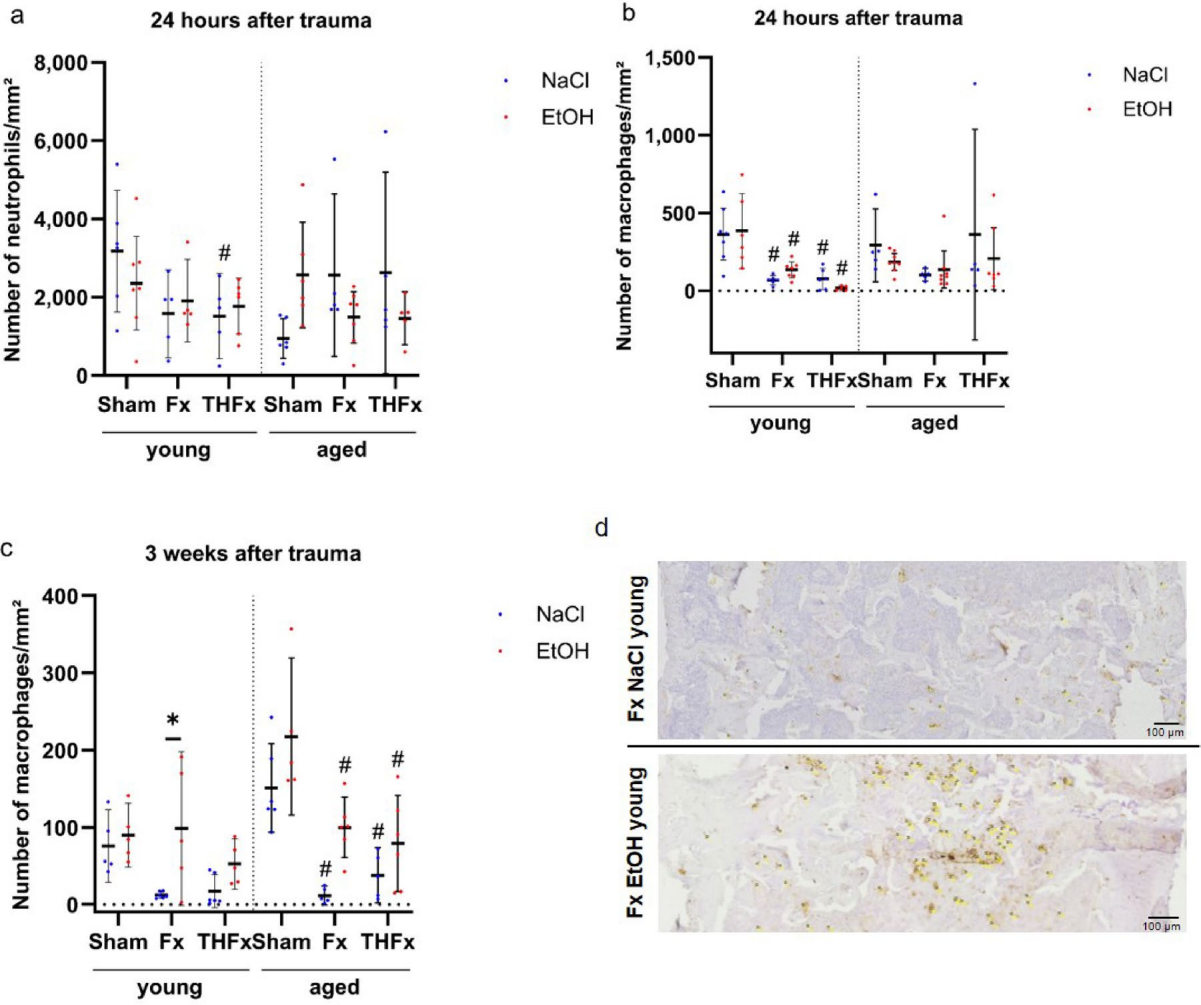
Local analyses of innate immune cells. The gavage with alcohol two hours before trauma had no effect on neutrophil (**a**) or macrophage numbers (**b**) 24 h after trauma. (**c**) After three weeks, drunken animals with an isolated fracture had more macrophages in comparison to the sober counterparts. Apart from that, only differences in comparison to the respective Sham groups were visible. (**d**) Significant changes in the numbers of macrophages after three weeks: stained structures, counted as macrophages in ImageJ, are marked with crosshairs and yellow numbers. Scale bar 100 μm, magnification 500-fold. # *p* < 0.05 vs. respective Sham. (A) *n* = 5–7, (B) *n* = 5–7, (C) *n* = 5–6.

For the number of neutrophils 24 h after trauma, significant changes were only observed in the young animals comparing the different traumata (*p* = 0.029) in comparison to the Sham groups as indicated (Fig. 3a). However, particularly in non-alcoholized old animals, neutrophil counts were very heterogeneous.

In the early phase, 24 h after intervention, alcohol gavage did not cause significant changes in the number of macrophages in the fracture gap or the representative area in the Sham groups in young or aged animals. Significant changes due to the trauma occurred only in the young mice (*p* ≤ 0.001) with reduced macrophage counts in the Fx and THFx groups compared to the respective Sham groups as shown in Fig. 3b. After three weeks, an acute alcohol consumption led to significant changes in the number of macrophages only in young mice (*p* = 0.003) with an overall trend in increasing macrophage counts (Fig. 3c, d). However significant changes were only observed in young mice in the isolated fracture group (adj. *p* = 0.015). In mice with advanced age, different traumata revealed significant changes (*p* < 0.001) with both Fx and THFx leading to a significant decrease in macrophages in the fracture gap when compared to respective Sham conditions.

### Bone and cartilage

**Fig. 4:**
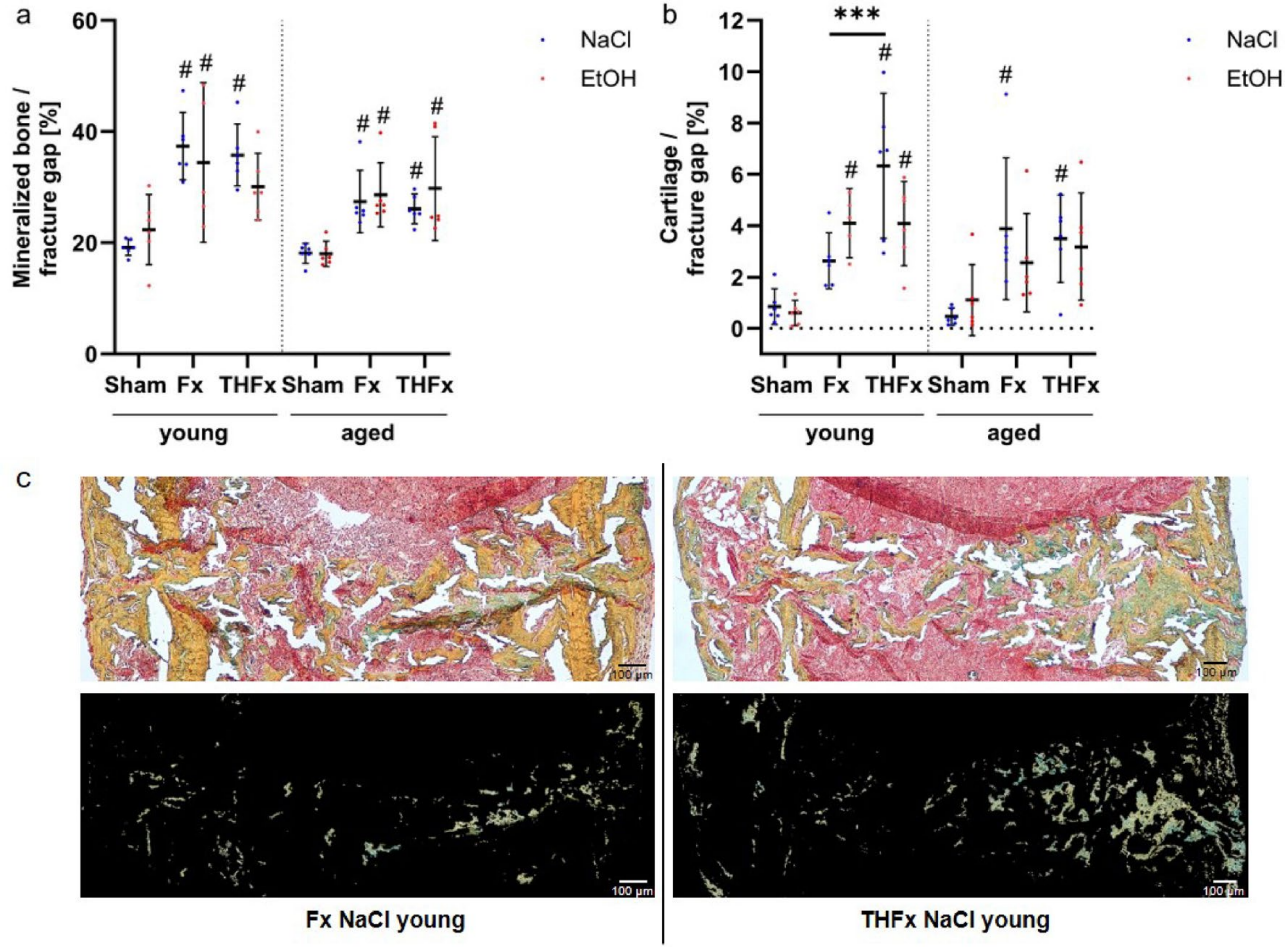
Local analysis of bone and cartilage. An acute alcohol intoxication did not lead to changes in mineralized bone (**a**) or cartilage (**b**) in the fracture gap, but a severe blood loss caused more cartilage in young sober mice than in the group with an isolated fracture (**b**,** c**). (**c**) Cartilage was measured as green stain in tissue slices stained with pentachrome. The upper image shows the original stained slice and the lower the selected green tissue for a representative animal of the indicated groups. Scale bar 100 μm, magnification 40-fold. *** *p* < 0.001 vs. indicated groups, # *p* < 0.05 vs. respective Sham. *n* = 5–6 per group.

Analyses of the mineralized bone in the fracture gap three weeks after trauma revealed no significant changes according to the type of gavage but between the different trauma groups (young: *p* < 0.001, aged: *p* = 0.002). More mineralized bone was observed in contrast to the corresponding Sham groups as displayed in Fig. 4a.

Cartilage production in the fracture gap showed a similar pattern. Three weeks after trauma (Fig. 4b), no significant differences on the cartilage content in the young and older groups were caused by single acute alcohol intoxication. However, in the young mice (*p* < 0.001), the different traumata showed a significantly increased share of cartilage with additional severe blood loss compared to an isolated fracture in young sober animals (adj. *p* < 0.001, Fig. 4b and c). This effect was not detectable in young drunken mice or the older ones. Apart from that, Fx and THFx procedures led to increased proportions of cartilage in the fracture gap compared to Sham conditions independent from age as indicated in in Fig. 4b.

### µCT analyses

**Fig. 5:**
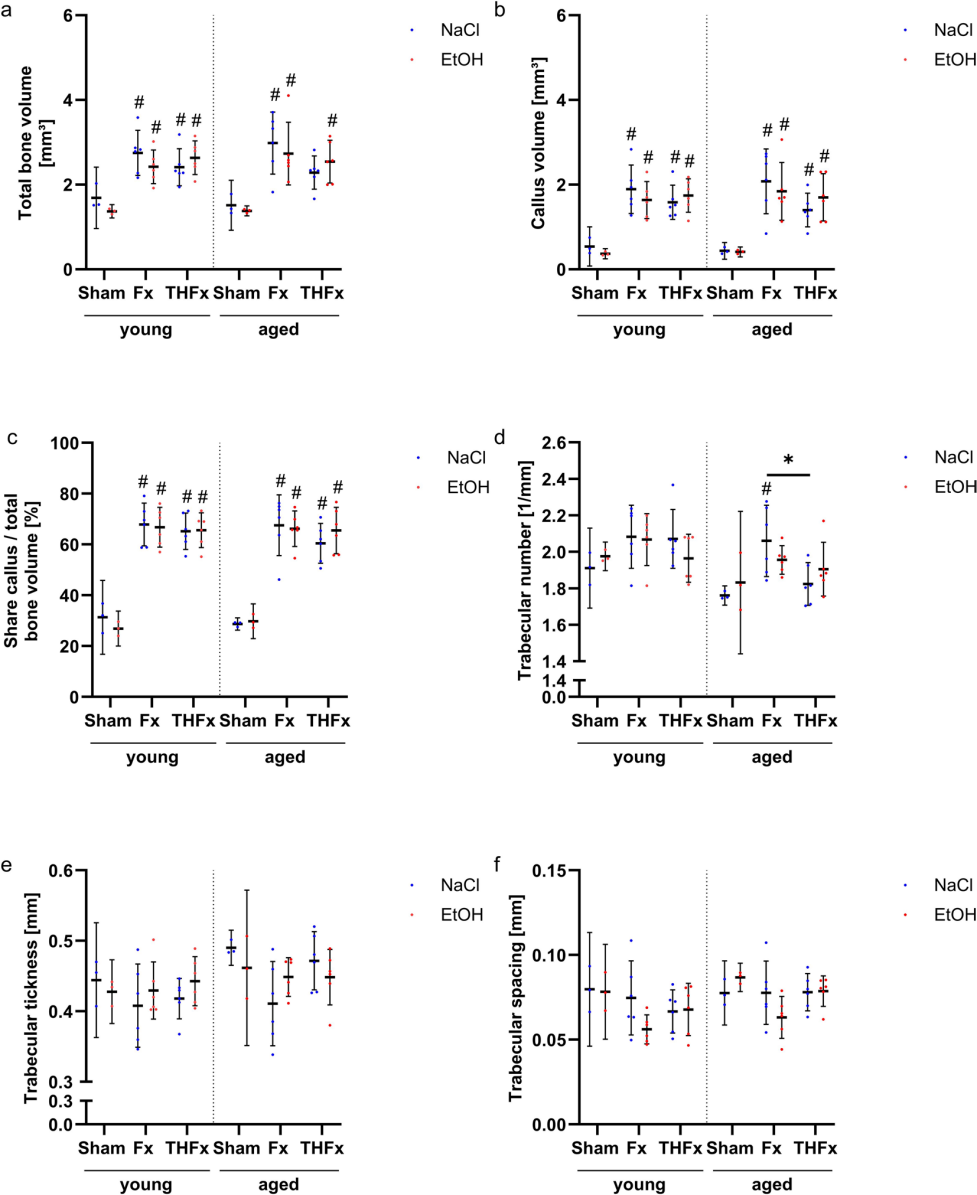
Analysis of µCT parameters. No effects of single binge alcohol consumption on fracture healing was detectable via µCT for the analyzed parameters total bone volume (**a**), callus volume (**b**), share callus/total bone volume (**c**), trabecular number (**d**), thickness (**e**) and spacing (**f**). With an additional blood loss, aged mice only showed a lower trabecular number in contrast to an isolated fracture (d). * *p* < 0.05 vs. indicated groups, # *p* < 0.05 vs. respective Sham. *n* = 3 for Sham; *n* = 6 for Fx and THFx.

Evaluation by µCT analyses of bone volume, callus volume, share callus/total bone volume (Fig. 5a, b, c) as well as trabecular number, thickness, and spacing (Fig. 5d, e, f) on femoral bones explanted three weeks after intervention showed no effect of alcohol administration in neither young, nor older mice.

However, the parameters total bone volume, callus volume, and the share of callus per total bone volume showed significant changes for the different trauma types (*p* < 0.001 for all) in comparison to the respective Sham groups (Fig. 5a, b, c).

The trabecular number also differed significantly between the trauma groups (*p* = 0.006) as shown in Fig. 5d. The additional hemorrhage led to significant less trabecular structures in comparison to the isolated fracture group solely in non-alcoholized elderly animals (adj. *p* = 0,013). Further, the aged Fx NaCl group also showed a significant rise in the trabecular number in contrast to the respective Sham mice.

### Biomechanical analyses

In the three-point bending test, no significant effects of a single dose of alcohol two hours before trauma were observed on biomechanical properties of the fractured bones three weeks after trauma in either young or old mice. Regarding the different trauma procedures, significant changes were found in the young animals for the maximum bending moment (*p* < 0.001), stiffness (*p* = 0.003) and elastic limit (*p* < 0.001), as well as in the aged animals for maximum bending moment and the elastic limit (both *p* < 0.001) compared to respective Sham groups as indicated in Supplementary Fig. 1a - c (online).

### Analyses of bone turnover markers in the plasma

**Fig. 6:**
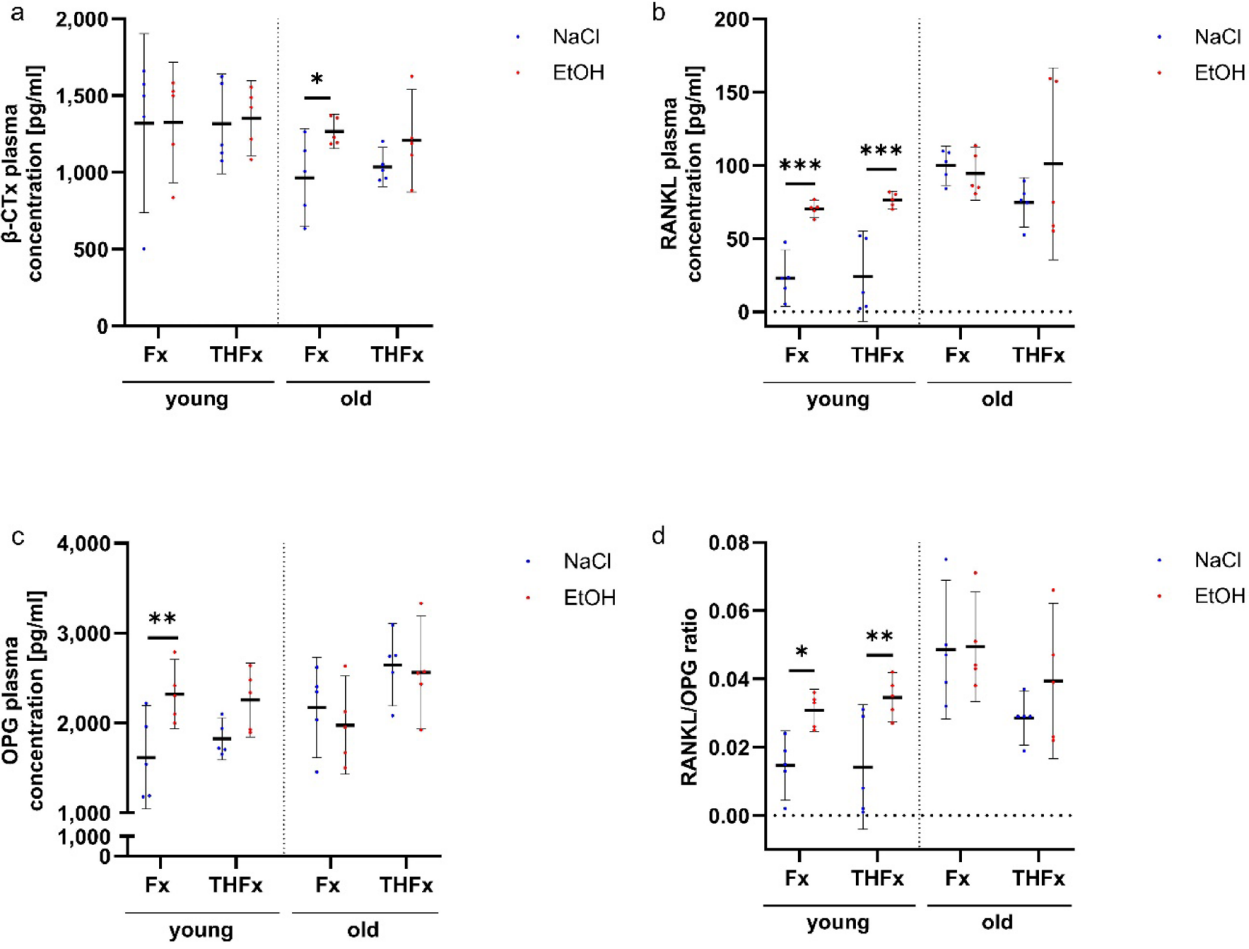
Systemically analyses of bone turnover markers via ELISA. (**a**) Aged drunken mice with an isolated fracture showed a higher concentration of β-CTx in the plasma than the sober counterpart. (**b**) The RANKL value was increased in both older Fx and THFx mice after alcohol intoxication in contrast to the NaCl groups. (**c**) OPG only rose in drunken Fx animals in comparison to the not drunken ones. (**d**) For the RANKL/OPG ratio, young mice with isolated fracture with and without severe bleeding had higher values than the sober animals. * *p* < 0.05, ** *p* < 0.01, *** *p* < 0.001 vs. indicated group; # *p* < 0.05 vs. respective Sham group. *n* = 5 per group.

Plasma β-CTx measured three weeks after trauma showed significant changes due to the gavage type only in the older animals (*p* = 0.017) as displayed in Fig. 6a. In these mice, acute alcohol intoxication resulted in higher β-CTx concentrations after isolated fracture compared to non-alcoholized animals of the same trauma group (adj. *p* = 0.029). This effect was not observed in young mice.

In contrast, a significant difference in the plasma concentration of RANKL three weeks after trauma was observed after alcohol gavage (*p* < 0.001) only in the young mice (Fig. 6b). In both young Fx (adj. *p* < 0.001) and THFx animals (adj. *p* < 0.001) an acute alcohol intoxication increased the RANKL concentration compared to the animals gavaged with only NaCl.

After three weeks, the OPG concentration also showed a significant change in response to the intoxication only in the young groups (*p* = 0.002) in form of a higher value in the isolated fracture group caused by an alcohol uptake two hours before trauma (adj. *p* = 0.009) as displayed in Fig. 6c.

In line, the RANKL/OPG ratio showed a significant difference for the alcohol intoxication in young (*p* < 0.001), but not in elderly mice (Fig. 6d). The acute alcohol intoxication two hours before trauma led to an increase of the RANKL/OPG ratio in the Fx (adj. *p* = 0.027) and THFx groups (adj. *p* = 0.006) in comparison to the sober animals.

## Discussion

The here presented study examined the long-term effects of a onetime binge alcohol intoxication on fracture healing in both young and aged mice, highlighting distinct physiological, systemic and cellular responses. Effects of age and trauma severity without alcohol intoxication have already been published^24,25^.

In the short-term, no immediate effects of the single acute alcohol consumption locally on the fracture site were observed in the histological evaluation after 24 h.

Concerning the long-term effects, alcohol intoxication caused systemically elevated levels of RANKL in young alcoholized animals with isolated fracture (Fx) and in combination with a severe blood loss (THFx) compared to sober mice. However, this increase was not observed in aged mice, which already showed higher baseline RANKL levels that were not further increased by alcohol. Despite more RANKL in the young drunken Fx and THFx groups, the number of osteoclasts did not change, but there was an increase in macrophages in the fracture group. Our study also observed more OPG in young alcoholized Fx mice compared to sober ones, with a similar trend in the THFx groups. This is consistent with the increase in the number of osteoblasts in the young intoxicated animals with isolated fracture compared to the unintoxicated mice after three weeks, which is also seen in the young Sham groups. As for RANKL, the aged sober mice already showed higher values of OPG that were not further elevated by alcohol intoxication. Moreover, the RANKL/OPG ratio was higher in young drunken Fx and THFx groups compared to sober mice, but no such effect was seen in the aged mice. This suggests that binge drinking caused a shift in the RANKL/OPG ratio towards more RANKL in the long term after fracture in young adult mice, aligning with the higher levels observed in sober aged mice. Apart from that, aged drunken Fx mice showed higher levels of β-CTx compared to their sober counterparts, indicating a higher rate of bone resorption in alcohol-treated aged mice. Despite these changes, there was no effect on bony bridging or biomechanical properties of the fracture, suggesting that alcohol did not impair the structural healing of the bone.

An additional severe blood loss showed negative effects only in sober mice but not in drunken ones. Severe hemorrhage led to a reduction in the trabecular number in non-alcoholized mice and an increase in cartilage formation, as well as more osteoclasts in the fracture gap in the young sober animals compared to an isolated fracture after three weeks. One reason for the lack of an aggravating effect of the additional severe blood loss after alcohol consumption could be that when removing blood, the organism also loses the alcohol, as well as cellular components such as immune cells and erythrocytes. Furthermore, the remaining blood alcohol was then subjected to a fourfold dilution with Ringer’s solution. This “dilution effect” also affects trauma-related pathogen-associated molecular patterns (PAMPs) and damage-associated molecular patterns (DAMPs), which have been demonstrated to impact regeneration.

In order to verify the results obtained here within the context of current scientific knowledge, a literature search was conducted for similar animal studies. This process revealed no studies analyzing single acute binge alcohol intoxication in combination with fracture healing. Therefore, the following discussion focuses on repeated acute binge drinking studies. Several studies have explored the detrimental effects of repeated acute binge alcohol consumption on fracture healing in various animal models. In a study of Lauing et al., male mice were administered three daily ethanol doses prior to a stabilized mid-shaft tibial fracture^9^. The results showed that 14 days post-fracture alcohol treatment led to a significant reduction in fracture callus volume, as well as biomechanical strength. Histologically, alcohol inhibited the formation of both bone and cartilage in the fracture gap, disrupting normal cartilage maturation. Volkmer et al. used adult male rats receiving binge alcohol cycles on three consecutive days with a break of 4 days over two weeks followed by unilateral closed femur fractures^14^. The study found that alcohol exposure significantly decreased biomechanical strength one and two weeks after fracture. Alcohol-treated animals moreover displayed less cartilage in the fracture callus altering the quality of fracture healing. The same alcohol regime was used in mouse models with tibial fracture showing similar effects as discussed in the following^10–13^. Obermeyer et al. observed that mice subjected to alcohol exposure prior to a tibial fracture had a significant impairment in biomechanical strength and callus volume upon µCT analysis, demonstrating that binge alcohol exposure prior to injury impairs fracture healing^12^. A follow-up study by Lauing et al. found that this binge alcohol regime resulted in decreased biomechanical strength of the fracture callus and histologically reduced cartilaginous callus formation^10^. In a study of Roper et al., the repeated binge alcohol intoxication model caused significantly reduced cartilaginous callus formation and hypertrophic chondrocyte area^13^. In the experiments conducted by Natoli et al., mice exposed to alcohol showed decreased external callus formation and reduced biomechanical stiffness, further indicating the negative impact of alcohol on bone healing^11^. The findings of the aforementioned studies demonstrate that exposure to binge alcohol on multiple occasions prior to injury results in impaired multiple phases of fracture healing, including delayed callus formation, disrupted endochondral ossification and compromised remodeling leading to reduced biomechanical properties. In contrast, one-time alcohol consumption prior to trauma used in our study was not able to identify any long-term effects on radiological, histological and biomechanical properties. Nevertheless, this single alcohol paradigm provides a more focused examination of the acute effects of alcohol on the fracture healing cascade. This model allows to isolate the immediate biological consequences of a single intoxication event without the compounding effects of chronic exposure. Our findings, including the altered RANKL/OPG ratio in young animals, suggest that even a single binge episode at the time of injury may prime the bone microenvironment in ways that could interfere with later stages of healing, despite minimal immediate structural changes. By comparing these different models, insight into the dose- and duration-dependent nature of alcohol’s effects on skeletal repair can be gained. The repeated binge model better represents the clinical reality of chronic heavy drinking, while the single binge model is more reflective of acute, incidental exposure that may occur around the time of injury in otherwise non-chronic users. This comparison also underscores the potential for even brief episodes of high alcohol intake to initiate molecular disruptions in the bone healing process, supporting the broader clinical message that alcohol consumption - even if episodic - can negatively influence fracture outcomes.

Having a closer look on the RANKL-OPG system in healthy bone metabolism, Cheng et al. found that chronic alcohol consumption for 12 weeks increased RANKL expression in both young (8 weeks) and old (72 weeks) male rats, with a more pronounced effect in older rats^15^. Systemic plasma OPG levels were higher in young rats after alcohol treatment, but no further increase occurred in older rats. Interestingly, OPG levels in sober old rats were increased compared to their younger counterparts. Alcohol also raised osteoclast numbers and reduced osteoblasts, especially in older rats. In our fracture study, single alcohol exposure raised plasma RANKL only in young animals, differing from bone expression patterns. The OPG results match with our findings, but our study observed more osteoblasts in young alcohol-treated mice (Sham and Fx) and no alcohol-induced changes in osteoclast numbers. The increase in the RANKL/OPG ratio three weeks after fracture in young alcohol-exposed animals found in our study suggests a shift in the local signaling environment towards increased osteoclastogenic potential. Although a corresponding change in osteoclast number or other markers of bone resorptive activity was not observed at this time point, this molecular change may reflect a dysregulation of the remodeling phase of fracture healing, which is tightly dependent on a balanced coupling between bone formation and resorption. The lack of changes in osteoclast number could indicate either a delay in the cellular response to the altered signaling, or a compensatory mechanism preventing osteoclast expansion despite the increased pro-osteoclastogenic drive. It is also possible that alcohol disrupts osteoclast function or lifespan in ways not captured by static cell counts or traditional resorption parameters. In terms of the implications for fracture repair, we propose that the elevated RANKL/OPG ratio in young alcohol-exposed animals, even in the absence of resorptive changes, could impair the coordination of remodeling necessary for proper callus maturation. The results suggest a potential for delayed or qualitatively compromised fracture healing, which may only become apparent at later stages or in combination with other comorbidities.

Despite careful planning of the animal study, there are a few limitations to consider. When designing the study, a calculation of the equivalent human age in the mouse was made with the result that 18–25 years in humans correspond to a mouse age of 17–24 weeks, and an aged human population of 60–70 years is reflected by 64–72 weeks in the mouse as already published^24^. However, this assumption is nowadays outdated for the aged group. Therefore, it must be assumed that our aged animals reflect more 40–45 year old humans than 60–70 years^25^. However, as this corresponds to the approximate average age of trauma patients admitted to an emergency department, this group is becoming increasingly important. Moreover, it must be noted that although the typical alcoholized trauma patient is male, as described above, and the study was therefore carried out on male mice, the phenomenon analyzed here also plays a crucial role in women. This is particularly relevant given that the underlying mechanisms in the female organism, especially in aged individuals prone to osteoporosis, may be significantly different from those in male individuals. Thus, female mice should also be included in such analyses in the future. Another limitation is the fact that only the fractured groups and not the Sham groups were analyzed in the ELISA measurements. Unfortunately, this cannot be performed retrospectively as the samples are no longer available for further analysis.

In conclusion, the here presented study showed that the single binge alcohol consumption in temporal proximity to a traumatic fracture, increased both the RANKL and OPG concentration systemically in young adult mice in the long-term similar to those in older, sober animals implying the systemic environment of bone remodeling to shift towards an aged phenotype. The therefore altered RANKL/OPG ratio may indicate a change in the regulation of bone turnover, which can lead to a dysregulation in the balance between bone formation and resorption, affecting bone metabolism in the long-term. Though a single binge drinking did not impair the local healing process of the fracture and initial healing seemed normal, systemic changes in RANKL und OPG may influence the bone quality and strength of the repaired bone over time.

## Methods

### Animal care

All animal experiments were approved by the Lower Saxony State Office for Consumer Protection and Food Safety (LAVES; animal experiment application number: 33.19-42502-04-17/2491), conducted according to the German Animal Welfare Law and the German Animal Welfare Experimental Animal Regulation and the ARRIVE guidelines were complied. Male C57BL/6J mice were purchased from Janvier Labs (Le Genest-Saint-Isle, France). Experiments began earliest after one week of acclimatization. Animals were housed in the central animal facility of the Hannover Medical School (MHH) under standardized conditions as described before^26^.

### Group distribution and gavage

With an age between 17 and 24 weeks (average 19.0 ± 1.6 weeks) for the young adult and an age of 64–72 weeks (average 66.4 ± 1.7 weeks) for the aged animals, mice were randomly assigned to one of the two end time points – 24 h or three weeks – and one of the three different operation groups: isolated fracture (Fx), fracture with additional trauma-hemorrhage (THFx) and Sham, each with and without acute alcohol intoxication (*n* = 8 for 24 h, *n* = 12 for three weeks per group, see Table 1). The initial weight was 29.1 ± 2.2 g for the young adult group and 33.0 ± 2.8 g in the aged group. The activity score was captured before gavage. Animals were orally gavaged with 3.5 g/kg bodyweight of either 35% ethanol or an equivalent volume of 0.9% NaCl two hours before operation. The volume was administered in two steps each with half the total volume and a break of 15 min. A red light lamp was placed in front of the cages so that the animals did not cool down due to the gavage, even if they were inactive or drowsy. Animals were allowed to move freely until surgery so that the distance to the heat source could be chosen independently.

**Table 1:** Number of animals per group used for the experiment.

Age	Gavage	OP	Sacrifice [*n*]		
			24 h	3 weeks	
				µCT and biomechanics	Histology
Young	NaCl	Sham	8	6	6
		Fx	8	6	6
		THFx	8	6	6
	EtOH	Sham	8	6	6
		Fx	8	6	6
		THFx	8	6	6
Aged	NaCl	Sham	8	6	6
		Fx	8	6	6
		THFx	8	6	6
	EtOH	Sham	8	6	6
		Fx	8	6	6
		THFx	8	6	6
Total			240		

### Surgical procedures

All surgical procedures were conducted under deep inhalation anesthesia with isoflurane. For analgesia, carprofen (5 mg/kg body weight) and butorphanol (1 mg/kg body weight) were injected subcutaneously before surgery and operation areas were locally anesthetized with prilocaine hydrochloride. Operation steps were performed as described before^24,25^. In brief, for the isolated fracture groups a commercially available external fixator (MouseExFix simple L 100%, RISystem, Davos, Switzerland) was implanted into the right femoral bone. Afterwards an osteotomy was performed at the diaphyseal part between the two middle pins with a 0.44 mm gigly wire saw, resulting in an osteotomy gap of 0.5 mm. For the THFx group, an additional severe blood loss was induced before fracture procedure. Thus, a catheter was implanted into the left femoral artery, the initial blood pressure was measured and blood was collected until a mean arterial blood pressure of 35 ± 5 mmHg was reached. This shock phase was maintained for 90 min, followed by reperfusion of four times the collected blood volume with a maximum of 2.4 ml body warm Ringer’s solution within 30 min. The Sham group was only implanted with external fixator and catheter, but no blood loss or fracture was performed. All surgical wounds were closed with continuous suturing for the muscles and single stiches for the skin. After operation, the animals were placed under red-light until full awakening. They were housed individually and health status was checked regularly according to an activity and lameness score. The activity score included six scoring points, from one reflecting healthy, active animals to six for moribund mice^24^. The lameness score included three points with a full weight bearing, lameness or no weight bearing^24^. For the first three days after surgery metamizole was added to the drinking water for continuation of analgesia postoperatively (200 mg/kg body weight). Carprofen (5 mg/kg body weight) and butorphanol (1 mg/kg body weight) were possibly injected indication depended.

### Harvesting procedure

Depending on the end time point, all animals were sacrificed 24 h or three weeks after surgery. First, cardiac exsanguination was performed with a heparinized syringe under injection anesthesia with ketamine (75 mg/kg body weight) and medetomidine (1 mg/kg body weight), finalized by cervical dislocation. Afterwards, the fractured femoral bone was harvested.

From animals sacrificed 24 h after surgery, femoral bones were used for histological analyses of cellular pattern in the fracture area. As these bones were not stable bridged, soft tissue and external fixator were not removed initially and samples were fixed for 24–48 h in 4% buffered formalin and transferred to 70% ethanol for longtime storage (*n* = 8). For the three week end time point, external fixator and soft tissue were immediately removed and bones stored depending on further analysis. Bones for µCT and biomechanical evaluation (*n* = 6) were stored at -20 °C and samples for histology were fixed 24–48 h in 4% buffered formalin and transferred to 70% ethanol for longtime storage (*n* = 6).

### Histological evaluation

#### Stainings

To analyze the cellular pattern in the fracture area 24 h after surgery, the external fixator was removed and bones were decalcified in 20% ethylenediaminetetraacetic acid (EDTA) solution for 14 days at 4 °C. Afterwards, ascending ethanol series were used to dehydrate the samples before embedding in paraffin. Via microtome (Reichert-Jung 2040, Leica Instruments GmbH, Nussloch, Germany) 3–4 μm thick slices were prepared, dried and before staining hydrated in descending alcohol series. Samples were stained as described before^25^. In brief, osteoclasts were stained via tartrate-resistant acid phosphatase (TRAP) with the following steps: 20 min in sodium acetate buffer (J.T.Baker, Deventer, Netherlands), two hours in TRAP staining solution containing Naphthol AS-MX Phosphate (# N4875, Sigma-Aldrich, Steinheim, Germany) and Fast Red TR Salt (# 368881, Sigma-Aldrich, Steinheim, Germany) and 1 min in Mayer’s Hemalum (Merck, Darmstadt, Germany). CD68 staining was used as a marker for macrophages. Therefore, the slices were transferred into 3% Hydrogen Peroxidase (#1.08597.1000, Merck, Darmstadt, Germany) Methanol solution in the dark, incubated with blocking solution (#ZUC007-100, Zytomed Systems, Berlin, Germany) in humid chamber for 30 min, followed by adding the primary antibody CD68 (E307V) Rabbit mAB (#97778, Cell Signaling Technology, Danvers, Massachusetts, USA) in a 1:400 dilution for one hour at room temperature. Afterwards, the SignalStain^®^ Boost IHC Detection Reagent (HRP, Rabbit) as secondary antibody (#30021200, Cell Signaling Technology, Danvers, Massachusetts, USA) was added for 30 min, followed by incubation with the DAB working solution for 10–15 min (DAB Substrate Kit, #DAB057, Zytomed Systems, Berlin, Germany). A counterstaining was performed by 1:100 Mayer’s Hemalum (Merck, Darmstadt, Germany). Additionally, neutrophil elastase (NE) staining was performed with 45 min in 1:10 diluted R-Universal Buffer (Aptum Biologics, Southampton, U.K.), 10 min in Hydrogen Peroxidase Block Solution (Abcam, Berlin, Germany), 60 min in 1:50 diluted primary antibody Neutrophil Elastase (#bs-6982R, Biozol, Munich, Germany), 30 min in HRP-Polymer Anti Rabbit (#ZUC032-006, Zytomed Systems, Berlin, Germany), 10 min in HRP-AEC Chromogen Substrate (#ZUC042-050, Zytomed Systems, Berlin, Germany) and 5 min in 1:100 diluted Mayer’s Hemalum (Merck, Darmstadt, Germany).

For histological evaluation after three weeks, bones were directly dehydrated using ascending ethanol series and embedded in Technovit 9100 (Heraeus Kulzer GmbH, Wehrheim, Germany) for evaluation of undecalcified samples. Slices of 5 µl thickness were cut via microtome (RM 2165, Leica Instruments GmbH, Nussloch, Germany) and dried before hydration in descending ethanol series including 2-methoxyethyl acetate (MEA). Samples were stained with Kossa-Safranin O (5 min 5% aqueous silver nitrate [Carl Roth, Karlsruhe, Germany] under light, 5 min fresh prepared Soda formol, 5 min 5% sodium thiosulfate, 5 min 1% Safranin-O [Merck, Darmstadt, Germany]) for evaluation of mineralized bone tissue (black color)). To analyze the cartilage amount (blue-green color), pentachrome staining (10 min in alcian blue [#3082.2, Sigma-Aldrich, Steinheim, Germany], 60 min in alkaline ethanol, 10 min in Weigert’s iron [#5192.1, Carl Roth, Karlsruhe, Germany] hematoxylin [#3816.1, Carl Roth, Karlsruhe, Germany], 15 min in Brilliant Croceine R [#1B109, Chroma/Waldeck, Muenster, Germany] - Acid Fuchsine O [#1B525, Chroma/Waldeck, Muenster, Germany], 20 min in phosphotungstic acid [1.00583.0100, Merck, Darmstadt, Germany] and 60 min in Safran du Gâtinais [#5A394, Chroma/Waldeck, Muenster, Germany]) was used. TRAP staining was performed as described above for evaluation of osteoclasts and osteoblasts. For the counting of osteocytes, slices were stained using the Safranin O /Light green protocol. For this, slices were incubated for 10 min in Weigert’s iron (#5192.1, Carl Roth, Karlsruhe, Germany) hematoxylin (#3816.1, Carl Roth, Karlsruhe, Germany), rinsed 10 min in flowing tap water and transferred to Light green solution (#Carl Roth, Karlsruhe, Germany) for 5 min. After short rinsing in 1% acetic acid (Merck, Darmstadt, Germany), the final staining step was incubation with 0.1% Safranin O solution (Carl Roth, Karlsruhe, Germany). Macrophages were also stained similar to the procedure for Paraffin-embedded samples, but the dilution of the primary antibody for Technovit samples was 1:200.

#### Analysis

The evaluation of bone and cartilage in the fracture gap or a representative area for Sham slices three weeks after trauma was performed as described before^27^. In brief, the BX41TF microscope (Olympus Tokyo, Japan) was used to take photos in a 40-fold magnification, and these photos were merged via Photoshop (Version 21.0). A software named “Image Filter Tool” was self-written in Java using the openCV library (version 4.1.0) for quantification of percentage of bone and cartilage in the representative areas^27^. Black tissue was evaluated as mineralized bone in the slices stained with Von Kossa/Safranin O. A blue-green color in the pentachrome staining reflected cartilage. Fixed threshold values were used to select the structures.

For evaluations on cellular level, pictures were taken using the VHX Digital microscope 7000 (Keyence GmbH, Germany) in different magnifications. Osteoclasts, osteoblasts and neutrophils were counted in a 300-fold magnification, whilst for macrophages the 500-fold magnification was chosen.

**Fig. 7:**
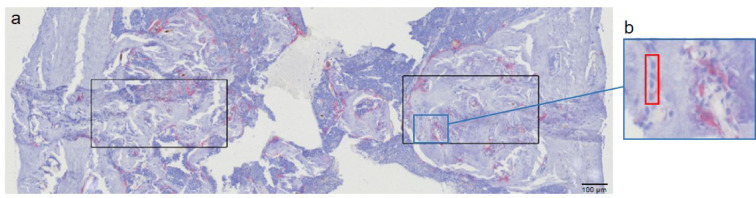
Evaluation of osteoblasts. Osteoblasts were counted in the fracture gap or an equivalent area in the Sham groups in TRAP-stained slices. (**a**) Two areas for counting were defined on the inner left and right cortical side within the medullary cave (black rectangles). (**b**) Violet, cubic cells arranged in lines with contact to bone are counted as osteoblasts (red rectangle). Scale bar 100 μm, magnification 300-fold.

TRAP-stained slices were used for manual counting of osteoclasts and osteoblasts. Osteoclasts were analyzed in the fracture gap or corresponding area for the Sham groups. For the THFx and Fx groups, the height of the area was based on the individual height and width of the fracture gap in. To determine the representative area in the Sham groups, the mean of the heights from the fracture (921.5 μm) was used as height, and the width was again selected individually depending on the thickness of the bone to be analyzed. This resulted in an average area of 1.82 mm². Active osteoclasts were defined as red colored, multinucleated cells with a ruffled border attached to the bone surface as already described^25^. Osteoblasts were counted in two areas with defined size on the left and right side of the inner bone (Fig. 7a, black areas). The areas had a defined height of 250 μm and width of 500 μm each, resulting in an average size of 0.125 mm^2^ for each area. In groups with fracture, the areas were placed in the fracture gap next to the sawed cortical structures. Representative areas were chosen for the Sham groups. In the TRAP staining, osteoblasts are colored violet/bluish and have a rather angular, cubic cell shape. The cells lie next to each other like a picket fence and never individually. They are always found in connection with bone tissue (Fig. 7b, red rectangle).

Osteocytes were counted manually in slices stained with Safranin O/Light green as already described^25^. Briefly, areas in the cortical bone on both sides were selected. The height of the areas was defined by the fracture area and the width by the cortical thickness. An equivalent setting was chosen in the Sham groups. Dark bluish, elongated or stellate cells in the cortical bone tissue were counted as osteocytes.

The analysis of macrophages was performed after staining of CD68 that made these cells appear as a brown structure. Macrophages have a round to oval shape, a foamy cytoplasm and an eccentrically located nucleus. Cells with connection to a bony surface were counted in the fracture gap or representative are in the Sham groups as described before^25^.

Neutrophils were counted semi-automatically after staining with NE between the cortical bone structures in the fracture gap or equivalent areas with the same height in the Sham groups as previously published^25^. In short words, the RGB mode in ImageJ was used to select neutrophils by individually setting thresholds for each slice as the color varied too much to find general values fitting for all images.

### µCT evaluation

For the µCT evaluation of the three week time point, all six animals from the Fx and the THFx groups, and three representative mice from the Sham group planned for later biomechanical evaluation were scanned in the Institute of Laboratory Animal Science of the MHH using Siemens Inveon µCT (Siemens AG, Munich, Germany). Calibration was performed using custom-made phantoms with defined densities (0, 50, 250, 750, 1200 mg HA/cm³; QRM, Möhrendorf, Germany) before every series of measurements. An effective pixel size of 8.17 μm was reached using the settings 360°, 360 steps, 7 s integration/position with 55 kV, 500 µA, filter 1 (0.8 carbon), high magnification and binning 1. For scanning, bones were defrosted under sterile conditions over a period of one hour at room temperature. Afterwards, samples were placed in a plastic tube and fixed by foam without tension to avoid movement during scanning. An area of interest of 1.2 mm in height between the two middle pin wholes was scanned reflecting the fracture gap. At the end of the scanning procedure the samples were stored at − 20 °C again for biomechanical analysis. Scans were analyzed using the Inveon Research Workplace 4.2 program. Bone parameters for volume of the total bone, as well as for the callus were revealed via the statistic tool. The percentage of callus/total bone volume was calculated by dividing the volume of the callus by the total bone volume in the fracture gap. Also, the parameters trabecular number, thickness and spacing were collected using the tool “bone morphometry”.

### Three-point bending test

All six bones per group were used for biomechanical evaluation. First, samples were transferred to Ringer’s solution and thawed for two hours at room temperature. The three-point bending test was conducted as described before in the Laboratory for Biomechanics and Biomaterials (LBB) of the Orthopedic Clinic of the MHH^26^. Briefly, the bones were placed in an anteroposterior position in the testing machine (MTS MiniBionix I, Model 858, Eden Prairie, Minneapolis, USA) and load was applied to the mid-diaphysis with a continuous lowering speed of 3 mm/minute without preload until termination criterion of 3 mm deflection was reached. These settings resulted in a complete structural failure in all samples. Load-deformation-curves were created to determine the parameters maximum bending moment [Nmm], stiffness [N/mm] and elastic limit [N].

### Evaluation of bone turnover markers in the plasma

The heparinized blood was centrifuged with 7.000 rpm at room temperature. Afterwards the plasma was collected and stored at -80 °C until further analysis. Enzyme-linked Immunosorbent Assays (ELISA) were performed on five randomly chosen animals for each Fx and THFx groups with and without ethanol administration three weeks after trauma (*n* = 5 per group). Analyses were made for β-CTx (#E-EL-M0372, Elabscience, Texas, USA), RANKL (#MTR00, R&D Systems, Minneapolis, USA) and OPG (#MOP00, R&D Systems) according to the manufacturers manuals. Additionally, the RANKL/OPG ratio was calculated from the measured values.

### Statistical evaluation

Statistical evaluation was done with the software GraphPad Prism version 9.0. Because of the complex study design, data of the young and aged animals were analyzed separately and not statistically compared to each other.

To evaluate the effect of alcohol on the physiologic parameters change in activity score (AS) and body temperature two hours after gavage, as well as the blood loss, animals were only analyzed for treatment with NaCl or EtOH. These data, as well as the change in the body weight were checked for normal distribution using the Anderson-Darling test. As dealing with ordinal data, the parameter change in AS two hours after gavage was evaluated non-parametric. For evaluation of body temperature and blood loss, t test for parametric and Mann-Whitney test for non-parametric data were conducted. The change in the body weight was analyzed as grouped data via Two-way ANOVA for young and aged mice (see below).

All other data were also divided into grouped data concerning alcohol treatment with the different traumata as subgroups. As published before^25^ and discussed with a preclinical data scientist, all of these data were considered parametric due to the small sample size. Analyzes for the grouped data of the young or aged animals were done via Two-way ANOVA to compare row (traumata) and column (alcohol treatment) factors (p value). Depending on occurring significant differences, further post-hoc tests between rows, columns or all subgroups were conducted (adjusted *p* value). Corrections for multiple comparisons were performed using the Tukey test for comparison between rows or all data and via Bonferroni test for comparison between columns.

All data are displayed as scatter plots with the values mean and 95% confidence interval. Statistical significance was assumed for *p* ≤ 0.05. Significant differences in comparison to the respective Sham group is shown in the graphs by an # and also described in the text but without mentioning the adjusted p value.

## Electronic supplementary material

Below is the link to the electronic supplementary material.

Supplementary Material 1.

Supplementary Material 2.

## Data Availability

The datasets analyzed during the current study are available from the corresponding author on reasonable request.
