# Supplementary material for: A single acute alcohol intoxication before fracture insult causes long-term elevated systemic RANKL and OPG levels in young adult mice
Source: Sci Rep. 2025 Jul 8;15:24423. doi: 10.1038/s41598-025-09240-3 (PMC12238551; doi:10.1038/s41598-025-09240-3)
Supplement: Supplementary file 1 — Supplementary Material 1. [file 41598_2025_9240_MOESM1_ESM.docx]

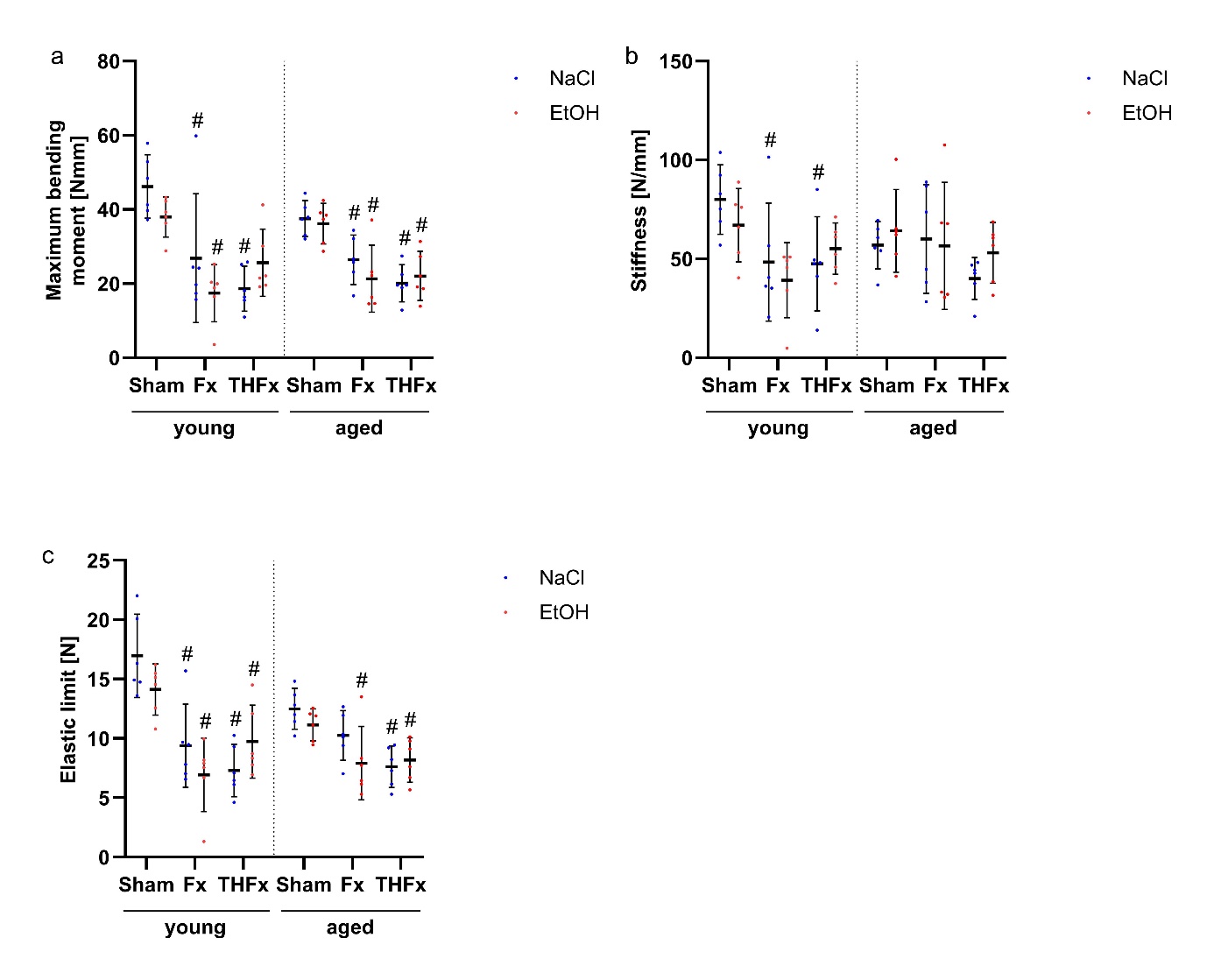


Supplementary Figure 1: Analysis of biomechanical parameters. No effects of single binge alcohol consumption or an additional blood loss was detectable in the biomechanical three-point bending test for the parameters maximum bending moment (A), stiffness (B) or elastic limit (C). Only differences against the representative Sham groups occurred. # p < 0.05 vs. respective Sham. n = 6 per group.
