# Supplementary material for: A single acute alcohol intoxication before fracture insult causes long-term elevated systemic RANKL and OPG levels in young adult mice
Source: Sci Rep. 2025 Jul 8;15:24423. doi: 10.1038/s41598-025-09240-3 (PMC12238551; doi:10.1038/s41598-025-09240-3)
Supplement: Supplementary file 2 — Supplementary Material 2. [file 41598_2025_9240_MOESM2_ESM.docx]

Supplemenetary Table 1: Group allocation of died, premature sacrificed and excluded animals.

| **Group** | **Notes** |
| --- | --- |
| **Died in experiment** | |
| Young EtOH THFx | Found dead 1,5h after EtOH gavage |
| Old NaCl THFx | Died in operation (reperfusion) |
| Young EtOH THFx | Died in operation after 35 minutes in TH |
| Old EtOH THFx | Died in operation (after reperfusion) |
| Old EtOH THFx | Died during operation due to unknown reasons |
| Old EtOH THFx | Died in operation during TH |
| Old EtOH Fx | Suddenly died 5 minutes after the surgery in wake-up phase |
| Young NaCl Fx | Suddenly died 10 minutes after the surgery in wake-up phase |
| Young EtOH THFx | Suddenly died 10 minutes after the surgery in wake-up phase |
| Old EtOH Fx | Suddenly died 30 minutes after the surgery |
| Old EtOH THFx | Died suddenly 50 minutes after operation, heavy breathing |
| Old EtOH Fx | Found dead in cage 1 hour after operation (probably due to EtOH gavage) |
| Old EtOH THFx | Found dead 2 hours after the surgery |
| Young NaCl THFx | Found dead 2.5 hours after surgery |
| Old NaCl THFx | Found dead 6 hours after the surgery, probably caused by TH due to unstable blood pressure during operation |
| Young NaCl THFx | Found dead on the next day after the surgery |
| Young NaCl THFx | Found dead in cage on day 4 |
| Old NaCl THFx | Found dead in cage on day 14 |
| **Premature sacrifice** | |
| Old EtOH THFx | Killed during surgery due to incorrect position of fixator |
| Old NaCl Sham | Killed during surgery due to incorrect position of fixator |
| Young EtOH Fx | Killed 1 hour after operation due to bad general condition |
| Old EtOH Sham | Squeezing after gavage, killing 1.5 hour after operation due to bad general condition |
| Young EtOH Fx | Killed 2.5 hours after operation due to bad general condition |
| Young NaCl THFx | Killed early on day 1 after operation due to bad general condition |
| Young NaCl Fx | In vivo mCT after 2 weeks showed dislocation of pins near hip (normal activity, no sign of lameness) |
| **Exclusion due to abnormal results** | |
| Young EtOH Fx | Not stable bridged 3 weeks after trauma (necessary for evaluation) |
| Young NaCl THFx | Not stable bridged 3 weeks after trauma (necessary for evaluation) |
| Young EtOH Sham | Not stable bridged 3 weeks after trauma (necessary for evaluation) |
| Old EtOH THFx | Not stable bridged 3 weeks after trauma (necessary for evaluation) |
| Old NaCl Fx | Abnormal bone healing |
| Old EtOH Fx | Abnormal bone healing |
| Old EtOH THFx | Abnormal bone healing |
| Old NaCl THFx | Loosening of the distal pin near to the knee resulting in a twisted bone healing |
| Old NaCl THFx | Huge cystic kidney |
